# Out‐of‐field dosimetry measurements for a helical tomotherapy system†

**DOI:** 10.1120/jacmp.v7i3.2212

**Published:** 2006-08-24

**Authors:** Chester R. Ramsey, Rebecca Seibert, Stephen L. Mahan, Dharmin Desai, Daniel Chase

**Affiliations:** ^1^ Thompson Cancer Survival Center Department of Radiation Oncology Knoxville Tennessee; ^2^ The University of Tennessee Department of Nuclear Engineering Knoxville Tennessee; ^3^ The University of Kentucky Department of Radiation Oncology Lexington Kentucky U.S.A.

**Keywords:** tomotherapy, intensity‐modulated radiation therapy, peripheral dose

## Abstract

Helical tomotherapy is a rotational delivery technique that uses intensity‐modulated fan beams to deliver highly conformal intensity‐modulated radiation therapy (IMRT). The beam‐on time needed to deliver a given prescribed dose can be up to 15 times longer than that needed using conventional treatment delivery. As such, there is concern that this delivery technique has the potential to increase the whole body dose due to increased leakage. The purpose of this work is to directly measure out‐of‐field doses for a clinical tomotherapy system. Peripheral doses were measured in‐phantom using static fields and rotational intensity‐modulated delivery. In‐air scatter and leakage doses were also measured at multiple locations around the treatment room. At 20 cm, the tomotherapy peripheral dose dropped to 0.4% of the prescribed dose. Leakage accounted for 94% of the in‐air dose at distances greater than 60 cm from the machine's isocenter. The largest measured dose equivalent rate was $1\times 10^{-10}$ Sv/s in the plane of gantry rotation due to head leakage and primary beam transmission through the system's beam stopper. The dose equivalent rate dropped to $1\times 10^{-10}$ Sv/s at the end of the treatment couch. Even though helical tomotherapy treatment delivery requires beam‐on times that are 5 to 15 times longer than those used by conventional accelerators, the delivery system was designed to maximize shielding for radiation leakage. As such, the peripheral doses are equal to or less than the published peripheral doses for IMRT delivery on other linear accelerators. In addition, the shielding requirements are also similar to conventional linear accelerators.

PACS number: 87.53.Dq

## I. INTRODUCTION

Over the past 10 years, the technology of radiation therapy has advanced considerably with the advent of intensity‐modulated radiation therapy (IMRT) and CT based image‐guided radiation therapy.^(^ ^1^ ^–^ ^8^ ^)^ While these technologies have the potential to deliver more conformal doses with a greater degree of accuracy, these techniques often require a larger number of monitor units (MUs) to deliver the prescribed dose. As the number of MUs required for treatment delivery increases, so does the primary beam leakage dose.^(^ ^7^ ^,^ ^8^ ^)^

Many studies have been published that investigated out‐of‐field doses for a multitude of linear accelerators.^(^ ^9^ ^–^ ^12^ ^)^ These studies all involved the measurement of peripheral doses from static beams at fixed gantry angles. More recently, out‐of‐field photon and neutron dose equivalent measurements were reported for IMRT delivery.^(^ ^13^ ^–^ ^15^ ^)^ Kry et al.^(^ ^14^ ^,^ ^15^ ^)^ reported dose equivalents measured in an anthropomorphic phantom that was irradiated for multiple conventional and intensity‐modulated treatment deliveries. They found that the photon and neutron doses varied depending on the manufacture of the linear accelerator, presumably due to differences in the manufacture of the accelerator head.

Helical tomotherapy is a rotational delivery technique that uses intensity‐modulated fan beams to deliver highly conformal IMRT.^(^ ^16^ ^–^ ^20^ ^)^ The treatment is delivered by a 6‐MV slip‐ring–mounted linear accelerator that continuously rotates about the patient. Like helical CT imaging, helical tomotherapy is delivered with the gantry and the couch in simultaneous motion. IMRT delivery is achieved by moving 64 individual collimators into and out of a narrow fan beam. The multileaf collimator (MLC) is binary, which means that each individual MLC leaf is either open or closed. The length of time that a leaf is open is proportional to the intensity of radiation allowed through that particular portion of the beam.

Depending on slice thickness (i.e., field size) and the number of rotations involved, the beam‐on time needed to deliver a prescribed dose can be up to 15 times longer than that needed for conventional treatment delivery. For example, the beam‐on time for a helical tomotherapy lung treatment delivered at 2 Gy per fraction typically ranges from 200 s to 300 s. The same treatment delivered with conventional (nonmodulated) parallel‐opposed fields typically requires 20 s to 60 s. Similarly, fixed‐gantry–based IMRT for prostate cases typically requires 50 s to 150 s of beam‐on time, while helical tomotherapy typical requires 300 s to 500 s.

Because of the increased beam‐on time, there is concern that this delivery technique has the potential to increase the whole body dose due to increased scatter and leakage.^(^ ^13^ ^–^ ^15^ ^)^ Low dose outside the treatment field can increase the risk of developing secondary cancers. This is of particular concern for patients with a life expectancy greater than 20 years. Furthermore, increased beam‐on time can lead to additional shielding requirements. According to the American Association of Physicists in Medicine (AAPM) IMRT Subcommittee, IMRT treatment delivery techniques that require a factor of 2 to 10 more MUs than conventional treatments should have the shielding evaluated.^(21)^

The purpose of this work is to directly measure out‐of‐field doses for a clinical tomotherapy system (HI‐ART, TomoTherapy, Inc., Madison, WI). The specific aims for this work are the following: (1) to measure tomotherapy peripheral doses for static fields and compare them with other published studies; and (2) to measure tomotherapy out‐of‐field doses for rotation treatment delivery and compare them with other published studies.

## II. MATERIALS AND METHODS

The HI‐ART tomotherapy system operates at a constant dose rate during treatment delivery (8.5−10 Gy/min at the isocenter).^(22)^ The dose delivered to the target volume in the patient depends on the X‐ray dose rate, primary collimator positions (i.e., slice thickness), the MLC delivery sequence (i.e., the delivery sinogram), pitch (i.e., amount of beam overlap per rotation), gantry rotation velocity, and number of total rotations.^(23)^ Unlike conventional linear accelerators, the tomotherapy system's output is not tied to the number of MUs. The number of “monitor units” displayed on the tomotherapy operator station is an arbitrary conversion of signal from the monitor chambers in the head of the accelerator. The treatment time is the primary measure of treatment duration, which is rigidly defined by the treatment‐planning system. The output of the accelerator is defined for planning purposes by a calibration file located in the treatment‐planning system. The tomotherapy inverse‐planning system uses this calibration file to calculate the treatment time.

### A. Static field peripheral dose

A miniature scanning water tank (Standard Imaging, Madison, WI) was used to measure profiles outside the treatment field. The scanning tank was designed to fit inside a helical tomotherapy treatment bore, and can measure profiles up to 50 cm in width and depth doses up to 20 cm deep with full backscatter. A 0.056 cm^3^ ionization chamber (A1SL, Standard Imaging, Middleton, WI) was used to measure longitudinal profiles (in and out of the bore), and a 1.91 cm^3^ ionization chamber (A17, Standard Imaging, Middleton, WI) was used as a reference probe. The scanning tank was leveled and positioned on the treatment couch with the tomotherapy isocenter placed at the surface of the water (85 cm source‐to‐surface distance). The electrometer bias was set to +300 V, and profiles were measured in 0.7‐mm intervals with a dwell time of 172 ms.

Measurements were taken for 6‐MV photon beams on a TomoTherapy HI‐ART (SN‐3) and a Varian 21EX (SN‐2838) for field sizes of $1.0\times 40.0\;{\text{cm}}^{2}$, $2.5\times 40.0\;{\text{cm}}^{2}$, and $5.0\times 40.0\;{\text{cm}}^{2}$ at depths of 1.5 cm, 5.0 cm, and 10.0 cm. These field sizes correspond to the slice thicknesses that are typically commissioned on the HI‐ART delivery system. The field sizes were defined using the primary jaw(s), and all MLC leaves were placed in the parked position during the measurements. Both Varian and TomoTherapy treatment couches were carefully inspected for sag before, during, and after data acquisition. The measured profiles were centered and normalized to the central axis dose. All reported distances are from the X‐ray field edge.

### B. Treatment delivery peripheral dose

The total out‐of‐field dose is dependent on the total beam‐on time required to deliver the prescribed dose. The peripheral doses to the patient are dependent on the treatment planning and delivery technique. As such, measurements must be taken using actual treatment delivery sequences.

**Figure 1:**
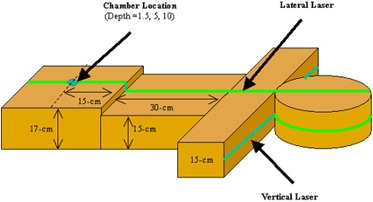
Water‐equivalent phantom used to measure peripheral doses. An ionization chamber was placed at depths of 1.5 cm, 5.0 cm, and 10.0 cm inside the phantom.

A test phantom was created out of three sets of water‐equivalent material (Fig. 1). The head of the phantom was a cylindrical piece of solid water 30 cm in diameter and 18 cm thick. The shoulders consisted of $15\times 55\;{\text{cm}}^{2}$ rectangular solid water stacked to an anterior‐posterior depth of 15 cm. The thorax of the phantom was two sets of $30\times 30\;{\text{cm}}^{2}$ solid water, with one set stacked to a height of 15 cm, and the second set stacked to a height of 17 cm.

In general, IMRT treatment delivery sequences with a high degree of modulation typically require a longer beam‐on time. For helical tomotherapy, head and neck treatments typically have one of the largest beam‐on times per gray of prescribed dose. A typical parotid‐sparing head and neck treatment plan was selected for measuring the out‐of‐field doses in the test phantom. This test plan extended 22 cm from the base of the skull to the bottom of the supra‐clavicular region. The planning target volume (PTV) included the gross tumor volume and the at‐risk lymph node chains with margin. The prescribed dose was 2 Gy per fraction to 95% of the PTV. The maximum cord dose was 70% of the prescribed dose, and 50% of both parotids was below 40% of the prescribed dose. The treatment required 336 s of beam‐on time with a slice thickness of 2.5 cm, a pitch of 1/3, and a modulation factor of 2.5, which are the standard planning parameters used at our institution.

A 0.6 cm^3^ cylindrical chamber (NEL‐2571) was placed in a custom‐milled piece of solid water and connected to an electrometer (35617EBS, Keithley, Cleveland, OH). Measurements were taken with the ion chamber placed at 10 cm, 15 cm, 20 cm, 25 cm, and 30 cm from the tomotherapy field edge. Measurements were taken at depths of 1.5 cm, 5.0 cm, and 10.0 cm. All readings are relative to the prescribed dose in the center of the tomotherapy field.

### C. In‐air scatter and leakage measurements

In‐air scatter and leakage doses were also measured to determine the required amount of shielding for radiation protection. The radiation dose equivalent was measured at various positions surrounding a tomotherapy system using a calibrated InoVision Model 451P ionization chamber (Cardinal Health, Inc., Dublin, OH), and absorbed dose was measured with a Standard Imaging A17 slice therapy ionization chamber. The 451P has a 300 cm^3^ collecting volume air ionization chamber that is pressurized to 8 atm (862 kPa). The A17 has a collecting volume of 1.91 cm^3^, a collecting volume length of 8.0 cm, a collector diameter of 2.4 mm, and a wall thickness is 3.3 mm and is specified to have a uniform response to within ±1.5% of average. Prior to use in this study, both chambers were calibrated by an accredited dosimetry calibration laboratory and are directly traceable to the National Institute of Standards and Technology standard.

Measurements with the A17 were taken with a build‐up cap that had a wall thickness of 1.5 cm placed over the body of the chamber. The chamber was connected to an electrometer (TomoTrometer, Standard Imaging, Middleton, WI), and measurements were acquired with a 300‐V bias. The American Association of Physicists in Medicine Task Group 51 dosimetry protocol was used to approximate the scatter and leakage absorbed dose.^(24)^ Since the beam quality $\left(k_{Q}\right)$ is not defined for in‐air scatter and leakage measurements, $k_{Q}$ was assumed to be equal to the beam quality for similar chambers in the calibration geometry (0.99). This $k_{Q}$ value is an approximation and should not be taken as having a strong basis in either theory or experiment. Leakage dose was measured using the A17 ion chamber placed at 30‐cm intervals from the front face of the TomoTherapy gantry.

The primary tungsten jaws and the tungsten MLC leaves together provide 23 cm of attenuation in the beam direction. Measurements that were taken with the primary jaws and all MLC leaves closed were considered to be leakage only. The combined scatter and leakage contribution was measured using the A17 and InoVision ion chambers placed at the same distance from the isocenter as the leakage measurements. A head and neck test plan was delivered with the solid water phantom (shown in Fig. 1) placed in the path of the beam to provide scatter.

### D. Patient measurements

Depending on the treatment‐planning parameters (slice thickness, pitch, degree of intensity modulation, etc.), the beam‐on time required to deliver 2 Gy can vary from 90 s to over 600 s. In addition to phantom measurements, scatter and leakage measurements were also taken during actual patient treatment deliveries. The dose levels were acquired for multiple patients to measure the level of radiation dose equivalent during typical helical tomotherapy treatment deliveries. The dose equivalent values were acquired with the 451P in integrated mode. Multiple measurements were taken for the same position, and measurements taken at equal distances laterally from the isocenter were averaged. Dose equivalent readings were scaled to the treatment time to determine the dose rate during treatment delivery. The dose equivalent rate in sieverts per second was calculated by dividing the dose by the beam‐on time for each measurement point.

## III. RESULTS AND DISCUSSION

### A. Static field peripheral dose

**Figure 2:**
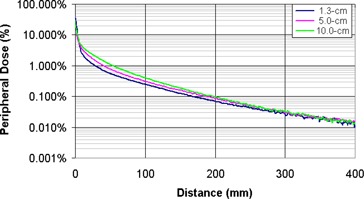
TomoTherapy peripheral dose distributions for a $2.5\times 40.0\;{\text{cm}}^{2}$ static beam at depths of 1.3 cm, 5.0 cm, and 10 cm

**Figure 3:**
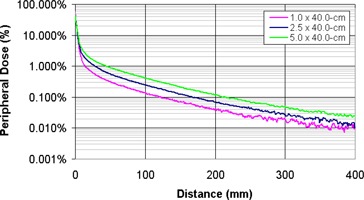
TomoTherapy peripheral dose distributions at a depth of 1.3 cm for $1.0\times 40.0\;{\text{cm}}^{2}$, $2.5\times 40.0\;{\text{cm}}^{2}$, $5.0\times 20.0\;{\text{cm}}^{2}$ static beams

A miniature scanning water tank was used to measure the peripheral dose for static 6‐MV fields on a Varian 21EX and a TomoTherapy delivery system. The measurements showed that the peripheral doses were independent of the measurement depth beyond 20 cm from the field edge (Fig. 2). The variation in peripheral closer to the field edge is due to changes in scattered dose at increasing depths. Figure 3 shows the peripheral dose variation at $d_{\max}$ for field sizes of $1.0\times 40.0\;{\text{cm}}^{2}$, $2.5\times 40.0\;{\text{cm}}^{2}$, and $5.0\times 40.0\;{\text{cm}}^{2}$. As the field size increases, so does the scatter dose contribution from the open field $\left(S_{c}\right)$. The peripheral dose variations with depth and field size measured in this study are consistent with previous studies.^(^ ^9^ ^–^ ^12^ ^)^

**Figure 4:**
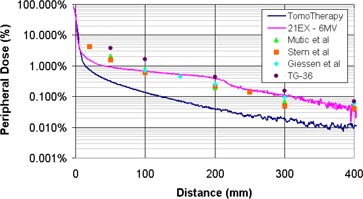
Comparison of peripheral dose distributions measured on a helical tomotherapy system and various conventional 6‐MV beams. The tomotherapy and 21‐EX measurements were taken with a $1\times 40\;{\text{cm}}^{2}$ field size. Mutic et al.^(9)^ data measured at $d_{\max}$ with the MLC set to $15\times 15\;{\text{cm}}^{2}$. Stern^(10)^ data measured at a depth of 5 cm with the MLC set to $10\times 10\;{\text{cm}}^{2}$. Giessen^(11)^ averaged measurements for 4‐ to 25‐MV linear accelerators with a $5\times 5\;{\text{cm}}^{2}$ field size. TG‐36 values were measured at $d_{\max}$ for a field size of $15\times 15\;{\text{cm}}^{2}$.

Figure 4 shows the peripheral dose measurements acquired in this study along with similar 6‐MV data from previously published studies. The 6‐MV measurements acquired on a Varian 21EX in this study agree well with previous studies.^(^ ^9^ ^–^ ^12^ ^,^ ^15^ ^)^ The discrepancy in the 21EX measurements at distances less than 10 cm from the field edge is due to the smaller field sizes that were used in this study. Mutic et al. measured peripheral doses at $d_{\max}$ with the MLC set to $15\times 15\;{\text{cm}}^{2}$.^(9)^ Stern measured peripheral dose at a depth of 5 cm with the MLC set to $10\times 10\;{\text{cm}}^{2}$.^(10)^ Giessen performed measurements for 4‐ to 25‐MV linear accelerators with a $5\times 5\;{\text{cm}}^{2}$ field size.^(11)^ The referenced TG‐36 values were measured at $d_{\max}$ for a $15\times 15\;{\text{cm}}^{2}$ field size.^(12)^ In this study, the 21EX measurements were made for comparative purposes with the TomoTherapy field sizes of $1.0\times 40.0\;{\text{cm}}^{2}$, $2.5\times 40.0\;{\text{cm}}^{2}$, and $5.0\times 40.0\;{\text{cm}}^{2}$.

The helical tomotherapy system used in this work was designed with the foreknowledge that the delivery technique inherently requires beam‐on times that are up to 15 times greater than those used in conventional treatment delivery. The primary tungsten jaws and the tungsten MLC leaves together provide 23 cm of attenuation in the beam direction. The MLC leaves were designed with an interlocking tongue‐and‐groove design to minimize leakage when the adjacent leaves are closed. In addition, the accelerator is shielded for leakage by a series of lead disks and a tungsten fixture. As such, the head shielding for the accelerating structure is greater than that in a conventional linear accelerator. This can be seen in the 3 to 11 times difference in peripheral dose between the conventional linear accelerator and the helical tomotherapy measurements in Fig. 4. In addition to head shielding, the system also has a beam stopper on the rotating gantry opposite the accelerator. The beam stopper consists of 13‐cm‐thick lead slabs that act as a counterweight and primary beam attenuator.

### B. Treatment delivery peripheral dose

During helical tomotherapy delivery, the treatment is continuously delivered on a slice‐by‐slice basis as the gantry rotates around the patient and the MLC modulates the intensity. The out‐of‐field peripheral doses from a tomotherapy treatment delivery will be greater than the open fixed‐field peripheral doses from the static fields reported in the previous section. To accurately model the peripheral doses delivered to patients during treatment, measurements were taken in a phantom during a simulated parotid‐sparing head and neck treatment.

**Figure 5:**
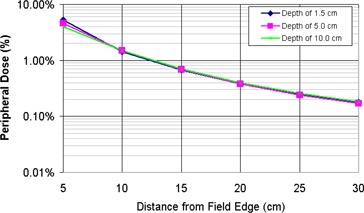
Out‐of‐field peripheral dose for a 336‐s (3360 MU) helical tomotherapy delivery. Measurements were taken in‐phantom at depths of 1.5 cm, 5.0 cm, and 10.0 cm. All distances are from the field edge, and the peripheral dose is normalized to the prescribed dose.

Out‐of‐field doses were measured for 6‐MV helical tomotherapy and parallel‐opposed delivery techniques with an ionization chamber placed at depths of 1.5 cm, 5.0 cm, and 10.0 cm inside a test phantom (Fig. 5). The measured peripheral doses were independent of the measured depth. The dose at 5 cm from the tomotherapy field edge was 4.6% of the prescribed dose, while the peripheral dose for parallel‐opposed delivery was only 1.5% of the prescribed dose. At 20 cm, the tomotherapy peripheral dose dropped to 0.4% of the prescribed dose, while

**Figure 6:**
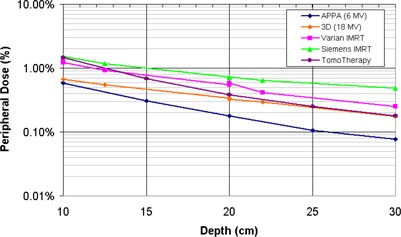
Comparison of measured dose equivalent for various treatment delivery techniques. All distances are from the field edge, and the peripheral doses are normalized to the prescribed dose. The out‐of‐field dose equivalents for the 18‐MV 3D, Varian IMRT, and Siemens IMRT delivery techniques were derived from Kry et al.^(^ ^14^ ^,^ ^15^ ^)^

The increased beam‐on time required for tomotherapy increases the out‐of‐field leakage dose to the patient. The amount of leakage dose is dependent on the transmission through the MLC leaves, the primary jaws, and the shielding around the accelerator/target. Even though helical tomotherapy delivery requires beam‐on times that are 5 to 15 times longer than those needed in conventional IMRT treatment delivery, the peripheral doses are equal to or less than the published peripheral doses for IMRT delivery on Varian and Siemens linear accelerators (Fig. 6).^(^ ^14^ ^,^ ^15^ ^)^ The less‐than‐expected peripheral doses for helical tomotherapy delivery are due to the increased shielding in accelerator design, the lack of flattening filter, and the narrow beamlets used to deliver the treatment.

### C. In‐air scatter and leakage measurements

**Figure 7:**
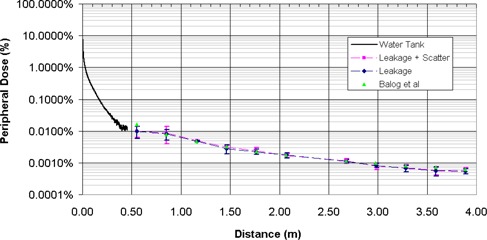
Out‐of‐field peripheral dose for a helical tomotherapy system. Water tank measurements taken for a $2.5\times 40.0\;{\text{cm}}^{2}$ open field at $d_{\max}$. Leakage and scatter measurements taken at $d_{\max}$ with an A17 Exradin Chamber. Leakage plus scatter measurements taken for a clinical delivery sequence with a slice thickness of 2.5 cm. Leakage‐only measurements taken with primary jaws and all MLCs closed. All distances are from the field edge, and the peripheral dose is normalized to the prescribed dose.

The relative contribution of scatter and leakage dose was measured in‐air at various distances from the machine isocenter using ionization chambers. A head and neck test plan was delivered with the solid water phantom placed in the path of the beam to provide scatter. The scatter and leakage contributions ranged from 0.01% to 0.0005% of the central axis dose at distances between 0.5 m and 4.0 m from the inferior border of the treatment field. The measured out‐of‐field dose contribution was predominately leakage, with 86% to 96% of the total measured dose at a point originating from head leakage. Scatter accounted for only 6% ± 4% of the in‐air dose at distances greater than 60 cm from the machine's isocenter. The scatter dose contribution decreases as the distance from the scatter (i.e., the patient) increases. Figure 7 shows the scatter and leakage measurements from this work compared with those of Balog et al., who also measured scatter and leakage on a helical tomotherapy system.^(25)^ The minor differences between the current study and that by Balog et al. is that the scatter and leakage measurements in this study were taken with a clinical delivery sequence. Balog et al. measured the combined scatter and leakage using an open $5\times 40\;{\text{cm}}^{2}$ field with all MLC leaves open. A clinical helical tomotherapy treatment delivery is composed of many small beamlets that are modulated using the binary MLC. As such, the scatter contribution for a clinical delivery will be smaller than a $5\times 40\;{\text{cm}}^{2}$ field for the same beam‐on time.

### D. Patient measurements

**Figure 8:**
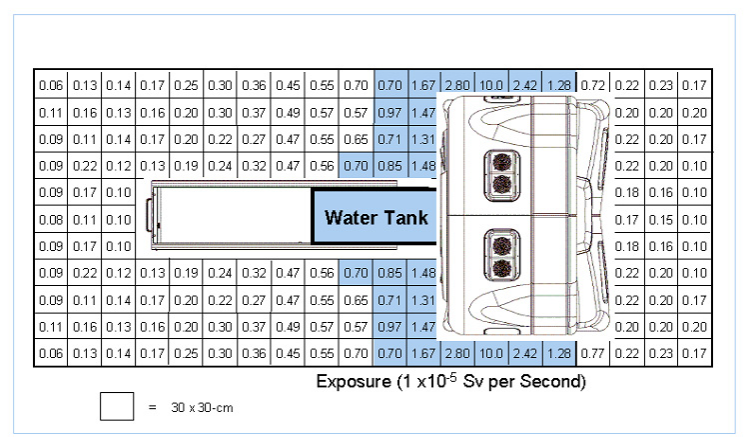
Dose equivalent rates measured at various locations around a tomotherapy system.

A total of 225 measurements additional were taken during helical treatment delivery for 25 patients at various positions around the tomotherapy gantry. The dose equivalent values in sieverts per second are shown in Fig. 8. The largest measured dose rate was $1\times 10^{-4}$ Sv/s in the plane of gantry rotation from head leakage and primary beam transmission through the beam stopper. The dose equivalent rate dropped to $1\times 10^{-6}$ Sv/s at the end of the treatment couch.

Typical beam‐on times for the HI‐ART system range from 120 s to 600 s, depending on the slice thickness, helical pitch, modulation factor, and target size. This is assuming an average beam‐on time of approximately 300 s and an average treatment slot of approximately 15 min. Given an 8‐h treatment day with four patients per hour, this yields 2.2 million seconds of beam‐on time annually. In addition to patient treatment, quality assurance measurements could add an additional 250 000 s to 500 000 s annually to the shielding requirements. The product of the dose equivalent values in Fig. 8 and the annual expected beam‐on time could be used to evaluate the shielding requirements for an existing vault or in the design of a new vault.^(^ ^25^ ^–^ ^31^ ^)^

Because of the beam stopper, there is a substantial decrease in the primary beam requirements for the tomotherapy system as compared to conventional linear accelerators. In most cases, the secondary barrier shielding for existing vaults should provide adequate primary beam shielding for a tomotherapy system. In general, the requirements for IMRT leakage shielding are greater than those for conventional accelerators because leakage is proportional to the beam‐on time. However, the leakage component in the tomotherapy is greatly reduced due to the 22 cm of tungsten shielding in primary jaws, the MLC, and head shielding.^(25)^

Based on the measured scatter and leakage values, a total of 3 to 5 tenth‐value layers of secondary beam shielding is required, depending on the room geometry, patient load, occupancy factors, and dose limits. In most cases, a HI‐ART system can be installed in an existing vault without the need for additional shielding.

## IV. CONCLUSIONS

The HI‐ART helical tomotherapy system requires beam‐on times that are 5 to 15 times longer than those required in conventional treatment delivery due to the fan‐beam delivery technique. However, the delivery system was designed to maximize the shielding for radiation leakage. As such, the peripheral doses are equal to or less than the published peripheral doses for IMRT delivery on other linear accelerators in most clinical radiotherapy applications. In addition, the shielding requirements are also similar to conventional linear accelerators.

## Supporting information

Supplementary Material
