# Supplementary material for: Out‐of‐field dosimetry measurements for a helical tomotherapy system
Source: J Appl Clin Med Phys. 2006 Aug 24;7(3):1–11. doi: 10.1120/jacmp.v7i3.2212 (PMC5722430; doi:10.1120/jacmp.v7i3.2212)
Supplement: Supplementary file 1 — Supplementary Material [file ACM2-7-01b-s001.doc]

**Out-of-Field Dosimetry Measurements for a Helical Tomotherapy System**

**Chester R. Ramsey, Ph.D.^1,2^**

**Rebecca Seibert, M.S.^2^**

**Stephen L. Mahan, Ph.D.^1^**

**Dharmin Desai, Ph.D.^3^**

**Daniel Chase, M.S.^1^**

[1] Thompson Cancer Survival Center, Department of Radiation Oncology, Knoxville, TN

[2] The University of Tennessee, Department of Nuclear Engineering, Knoxville, TN

[3] The University of Kentucky, Department of Radiation Oncology, Lexington, KY

### Chester R. Ramsey, Corresponding Author

Thompson Cancer Survival Center

*Department of Radiation Oncology*

*1915 White Ave.*

### *Knoxville, TN 37916*

*Phone: 865-541-3161*

*Fax: 865-541-1801*

*Email: cramsey@utk.edu*

This work was presented at the 47^th^ annual meeting of the

American Association of Physicists in Medicine
